# Cardiopulmonary point-of-care ultrasound for critical care

**DOI:** 10.1016/j.clinme.2026.100624

**Published:** 2026-07-20

**Authors:** Hatem Soliman Aboumarie

**Affiliations:** a Department of Cardiothoracic Anaesthesia and Intensive Care, Harefield Hospital, Royal Brompton and Harefield Hospitals, London, UK; b School of Cardiovascular, Metabolic Medicine and Sciences, King’s College London, UK

**Keywords:** Point-of-care ultrasound (POCUS), Critical care echocardiography (CCE), Shock, Haemodynamic assessment, LVOT VTI, Preload responsiveness, Lung ultrasound, Systemic venous congestion, Mechanical circulatory support, Cardiopulmonary interactions

## Abstract

Point-of-care ultrasound (POCUS) has emerged as an essential bedside tool for the rapid assessment and management of critically ill patients. This review outlines the evolution of POCUS from a focused, rule-in/rule-out modality to an advanced, integrative diagnostic approach capable of supporting complex haemodynamic evaluation. Technological advances, including handheld devices and artificial intelligence, have enhanced accessibility, diagnostic accuracy, and training efficiency. In the intensive care setting, echocardiographic interpretation must be contextualised within dynamic physiological variables such as preload, ventilation, vasoactive support, and metabolic status. POCUS plays a pivotal role in differentiating shock phenotypes—cardiogenic, distributive, hypovolaemic and obstructive—by integrating structural and functional cardiac assessment with lung and systemic venous ultrasound findings. Key haemodynamic parameters, including left ventricular outflow tract velocity time integral (LVOT VTI) and Doppler-derived indices, enable evaluation of cardiac output and fluid responsiveness, while dynamic tests such as passive leg raising further refine management decisions. The review also highlights the role of POCUS in assessing hypoxaemia through combined cardiopulmonary evaluation and introduces the ʻPOCUS pyramid’ framework, integrating cardiac, pulmonary, and venous domains into a unified physiological model. Ultimately, when performed by appropriately trained clinicians, POCUS and critical care echocardiography enhance diagnostic precision, guide therapy, and improve bedside decision-making while recognising inherent limitations and the need for structured training and governance.

## Graphical Abstract

**Figure:**
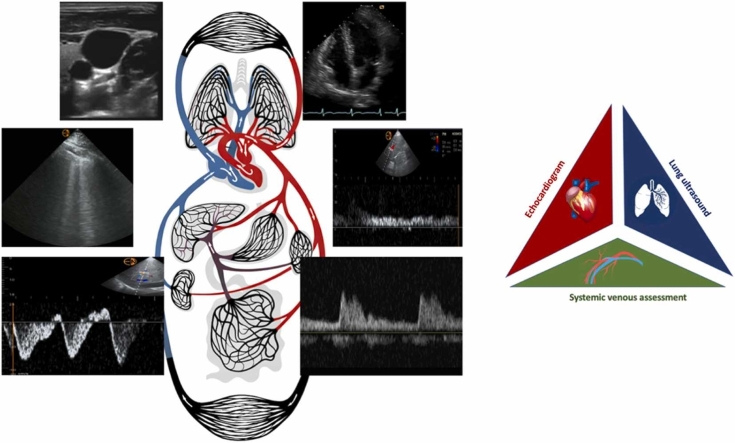
This central illustration summarises the POCUS pyramid which integrates the three pillars of haemodynamic POCUS (cardiac, lung and systemic venous congestion).

## Introduction and definitions

Point-of-care ultrasound (POCUS) represents the acquisition, interpretation, and immediate clinical integration of ultrasonographic imaging performed directly by the treating clinician. This modality is distinct from traditional ʻconsultative’ echocardiography, which is performed and interpreted by a separate team—often a sonographer and a physician—within an echocardiography laboratory at the request of the treating physician. POCUS has become an indispensable tool in managing cardiac patients, providing real-time, bedside imaging that enables rapid, informed clinical decision-making.

Over the last decade, POCUS has evolved from a limited ʻrule-in or rule-out’ examination for novices into an advanced diagnostic approach capable of supporting complex cardiovascular assessments when performed by experienced practitioners. Contemporary practice spans a spectrum from basic to complex, depending on the clinical setting, indication, and the clinician's training. By integrating cardiac ultrasound with lung ultrasound and systemic venous congestion assessments, clinicians can achieve a comprehensive evaluation of cardiopulmonary interactions and volume status.^1^

This review summarises the contemporary applications of POCUS in critical care. Following an overview of the evolution of POCUS, terminology, technological developments and training considerations, we discuss the physiological principles underpinning its use in critically ill patients. We then review the role of POCUS in differentiating shock phenotypes, assessing haemodynamics and preload responsiveness, and evaluating hypoxaemia through integrated cardiopulmonary ultrasound. Finally, we present the proposed POCUS pyramid as a framework for multimodal bedside assessment and discuss the practical considerations, limitations and future directions of POCUS in critical care practice.

## Technological evolution and diagnostic impact

The advent and rapid expansion of POCUS have been driven by the transition from large, cumbersome equipment to miniaturised, pocket-sized devices. Handheld echocardiography (HHE) devices, which appeared over two decades ago, have undergone dramatic technological advances in resolution and portability. Modern units are lightweight, self-contained and often engineered within the ultrasound probe itself, using smartphones or tablets as displays.^2^

Standard capabilities now include B-mode grayscale, M-mode, colour Doppler and increasingly, continuous wave Doppler. Furthermore, the incorporation of artificial intelligence (AI) has enabled interactive guidance for image acquisition, accelerated learning for novices, and automatic measurement of basic echocardiographic parameters. Comparative studies have demonstrated the incremental value of POCUS over traditional physical examinations. For example, research showed that the area under the receiver operating characteristic (ROC) curve for cardiovascular diagnosis was 0.70 for physical examination alone, but jumped to 0.92 for POCUS.^3^ A consensus statement published by the American Society of Echocardiography over a decade ago found that POCUS provided accurate assessment for multiple echocardiographic findings including left ventricular (LV) enlargement (six studies), LV hypertrophy (eight studies), LV systolic function (19 studies), left atrial (LA) enlargement (nine studies), right ventricular (RV) enlargement (three studies), RV systolic function,^4^ pericardial effusion (11 studies), and inferior vena cava (IVC) size (two studies).^5^

The primary indications for POCUS include:

1) Emergent or urgent need for clinical evaluation: for rapid triage and clinical management directed by the treating clinician.
2) Ongoing critical illness management: performance of frequent serial follow-up assessments.
3) Cardiovascular disease screening: most often where there is unavailability of standard echocardiographic services, as in underserved or remote populations where standard platforms are unavailable (e.g., rural and remote applications), or for specific population screening (e.g., athletes, elderly at risk for valvular heart disease, etc).

## Terms and definitions for cardiopulmonary sonography

Advanced practice, known as critical care echocardiography (CCE), demands skills beyond basic POCUS, including the standard use of Doppler, transoesophageal echocardiography (TOE) and potentially advanced techniques like 3-D imaging, strain, and contrast. Certification in CCE is available for those meeting specific competence criteria.^4^ (Table 1).

**Table 1:** Definitions and terminology of cardiac ultrasound in cardiovascular emergencies and critical care adapted from Soliman-Aboumarie *et al*.^1^

Term	Definition	Applications
Emergency echocardiography	Comprehensive echocardiographic examination is performed in an emergency setting in the assessment of patients with unstable CVDs by an operator fully trained in echocardiography, which acquires a well-defined data set.	Full assessment of cardiac morphology and function
FoCUS	A specific type of POCUS applied to the heart, as an extension of the clinical examination, by an operator not necessarily trained in comprehensive echocardiography, but appropriately trained in FoCUS, usually responsible for decision-making and/or treatment.	Rapid assessment of global left and RV function, estimating volume status, detecting pericardial effusion/tamponade
POCUS	A goal-oriented, bedside, limited ultrasound examination, extending physical examination performed in any body structure and environment with a predefined limited protocol. Often involves cardiac and non-cardiac assessments (i.e., lung, venous, neurological and abdominal ultrasound). Can be performed on a basic to advanced operator level.	Bedside multi-organ ultrasound assessment to guide clinical bedside decision-making
Targeted (goal-oriented) echocardiography	A targeted echocardiographic examination performed by an operator fully trained in echocardiography.	Attempting to obtain an answer to a specific, often critical and frequently complex clinical dilemma
CCE	CCE involves comprehensive assessment using both standard and advanced echocardiographic techniques to evaluate cardiac function, haemodynamics, assessing preload, afterload, and cardiac output as well as pulmonary and SVR. This requires advanced-level training in CCE.	Monitoring cardiac function, haemodynamics, treatment response in shock, ARDS, guiding the insertion, monitoring, troubleshooting and weaning from MCS devices
fTOE:	This involves inserting an ultrasound probe into the oesophagus to obtain detailed images of the heart by a fully trained echocardiographer. fTOE provides superior image quality, especially in critically ill patients where transthoracic windows are inadequate. This requires comprehensive training on TOE.	It is particularly useful in detecting aortic dissection, endocarditis, for guiding cannulation and troubleshooting from ECMO and MCS, and also useful during cardiopulmonary resuscitation.

FoCUS, focused cardiac ultrasound; POCUS, point of care ultrasound; CCE, critical care echocardiography; ARDS, acute respiratory distress syndrome; ECMO, extracorporeal membrane oxygenation; fTOE, focused transoesophageal echocardiography; MCS, mechanical circulatory support; SVR, systemic vascular resistance.

## Pathophysiologic factors affecting cardiopulmonary sonography in critical care

**Fig. 1:**
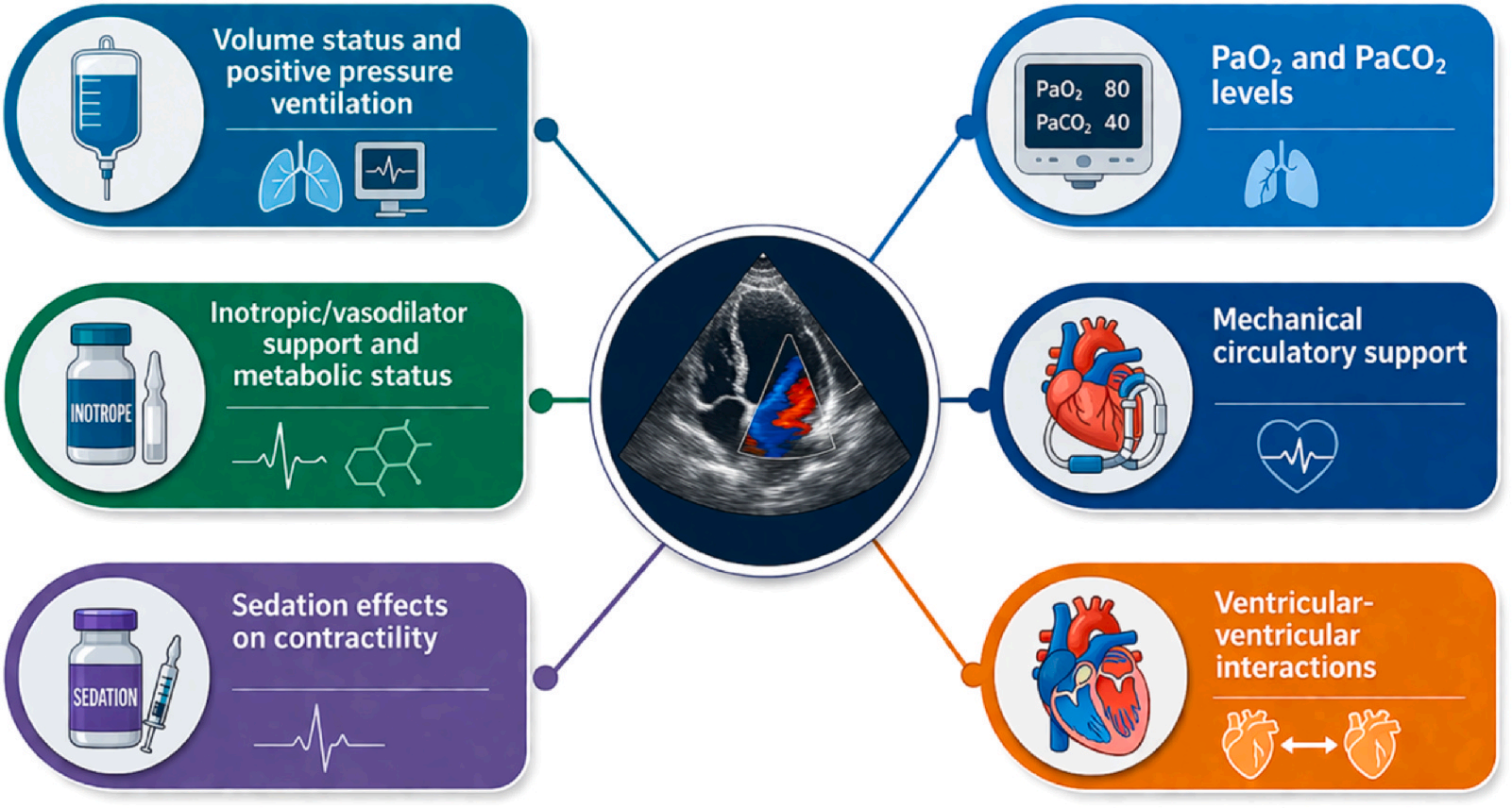
Key considerations which may influence interpretation of echocardiographic findings in ICU patients (CCE).^6^

In the intensive care unit (ICU), interpretation must account for highly dynamic factors. Cardiac structure and function are frequently altered by changes in intravascular volume, positive pressure ventilation, and the use of inotropic or vasodilator therapy. Metabolic derangements, oxygenation levels and the effects of sedative agents also modify myocardial performance. Furthermore, mechanical circulatory support (MCS) and the divergent responses of the right and left ventricles to interventions add layers of complexity (Fig. 1).

## The role of POCUS in shock states

Shock is a life-threatening circulatory failure resulting in insufficient cellular oxygen delivery. Because clinical assessment alone can be challenging—as all shock types may present with hypotension and impaired perfusion—POCUS is invaluable for differentiating between cardiogenic, hypovolaemic, distributive, and obstructive etiologies.

### Cardiogenic shock

This syndrome results from primary cardiac pump failure. Historically defined by sustained hypotension (SBP <90 mmHg) and end-organ hypoperfusion (lactate >2.0 mmol/L), contemporary frameworks now emphasise imaging and biochemical evidence of dysfunction over rigid pressure thresholds. However, latest guidelines have moved away from mandating low blood pressure as a criterion for shock.^8^

#### Echocardiographic features

The most frequent cause is severe LV systolic dysfunction following myocardial infarction (MI). Typical findings include a globally hypocontractile LV with regional wall motion abnormalities. Approximately 10–15% of post-MI cases involve mechanical complications such as:

• Acute mitral regurgitation (MR) from papillary muscle rupture or functional tethering.
• Ventricular septal defect (VSD).
• Free wall rupture with tamponade.

#### Haemodynamic assessment

Ejection fraction (EF) is commonly used but remains highly sensitive to preload, afterload and heart rate. Consequently, estimating cardiac output (CO) via Doppler is often more informative. This involves measuring the LVOT area and multiplying it by the LVOT velocity time integral (VTI). A normal LVOT VTI is typically >18–20 cm.

Diastolic function is assessed by combining pulsed-wave Doppler of transmitral inflow with tissue Doppler imaging (TDI) of the mitral annulus, as these techniques provide complementary information. Transmitral inflow velocities reflect the pressure gradient between the left atrium and left ventricle. The E wave represents early passive ventricular filling immediately after mitral valve opening, while the A wave represents late ventricular filling generated by atrial contraction. In contrast, tissue Doppler velocities measure the movement of the mitral annulus and therefore reflect intrinsic myocardial relaxation and longitudinal ventricular function rather than blood flow. The e′ velocity corresponds to early diastolic annular relaxation and is a relatively preload-independent marker of left ventricular relaxation, whereas the a′ velocity reflects annular motion during atrial contraction and contributes to assessment of late diastolic function. Because transmitral E velocity is influenced by both ventricular relaxation and left atrial pressure, while e′ primarily reflects myocardial relaxation, the E/e′ ratio provides an estimate of left ventricular filling pressure. An E/e′ ratio <8 generally suggests normal filling pressures, whereas a ratio >14 is consistent with elevated left ventricular filling pressures; intermediate values should be interpreted alongside other echocardiographic parameters.^9^

### Distributive shock

Characterised by profound vascular tone abnormalities, distributive shock often presents with preserved or increased cardiac output but inadequate tissue perfusion.

• Septic shock: Driven by widespread vasodilation and capillary leak. POCUS identifies the dominant derangement (hypovolaemia, vasoplegia, or myocardial dysfunction).
• Sepsis-induced cardiomyopathy: Occurs in 10–70% of septic patients.^10^ Features include acute onset (reversible within 7–10 days), global biventricular dysfunction, and LV dilation. Global longitudinal strain (GLS) is a more sensitive marker than LVEF for detecting this impairment.^10^

### Hypovolaemic shock

Caused by a critical reduction in intravascular volume (haemorrhage, gastrointestinal loss, or third-space sequestration), this leads to reduced preload. POCUS identifies a small IVC (<2.1 cm) and hyperdynamic, small cardiac chambers. In hypovolaemia also the flow in systemic veins shows evidence of normal right atrial pressure and absence of systemic venous congestion as well as predominant A profile on lung ultrasound.^11^, ^12^

#### Preload responsiveness

Predicting preload responsiveness is crucial, as only 40–70% of shock patients benefit from volume expansion.^13^ Dynamic indices are superior to static measures:

• IVC distensibility index: In mechanically ventilated patients, a value greater than 18% predicts responsiveness^14^
• *IVC distensibility index = (IVC_max − IVC_min) / IVC_min × 100%*
• SVC collapsibility index: A threshold of greater than 36% via TOE indicates responsiveness.^15^
• *SVC collapsibility index = (SVC_max − SVC_min) / SVC_max × 100%*
• Passive leg raising (PLR): Provides a reversible autotransfusion of 300–500 mL, making it reliable for assessing preload responsiveness in spontaneously breathing patients and patients with atrial fibrillation.^16^

### Obstructive shock

#### Cardiac tamponade

Accumulating pericardial fluid increases intrapericardial pressure, leading to external cardiac compression. Key POCUS findings include:

• early diastolic collapse of the RV free wall
• late diastolic collapse of the RA
• dilated IVC with <50% respiratory variation
• exaggerated ventricular interdependence (septal bounce)
• respiratory variation in transvalvular flows (>40% increase in tricuspid inflow; >25% decrease in mitral inflow on inspiration).

#### Pulmonary embolism (PE)

Acute high-risk PE causes sudden RV pressure overload. Echocardiographic markers include:

• McConnell’s sign: RV free wall akinesia with preserved apical contractility
• 60/60 Sign: A combination of pulmonary acceleration time (PAT) <60 ms, often accompanied by a mid-systolic notching of the right ventricular outflow tract Doppler envelope, indicating increased pulmonary vascular impedance, together with a tricuspid regurgitation (TR) pressure gradient <60 mmHg. This pattern suggests acute pulmonary hypertension, most commonly due to acute pulmonary embolism, where a sudden increase in pulmonary vascular resistance shortens pulmonary ejection despite the right ventricle being unable to generate markedly elevated systolic pressures. The 60/60 sign helps distinguish acute pulmonary embolism from chronic pulmonary hypertension, in which pulmonary acceleration time is also shortened but the TR gradient is typically >60 mmHg.
• RV/LV ratio: basal diameter ratio >1.0
• TAPSE: reduced tricuspid annular plane systolic excursion (<16 mm)
• a thrombus could be seen in a deep vein in the calf or groin

#### Tension pneumothorax^17^

This life-threatening condition impairs venous return via high intrapleural pressure. Lung POCUS findings include:

• absence of lung sliding (seashore sign becomes barcode/stratosphere sign on M-mode)
• absence of B-lines and presence of prominent A-lines
• lung point: the specific location where normal lung sliding reappears

#### Dynamic LVOT obstruction

**Fig. 2:**
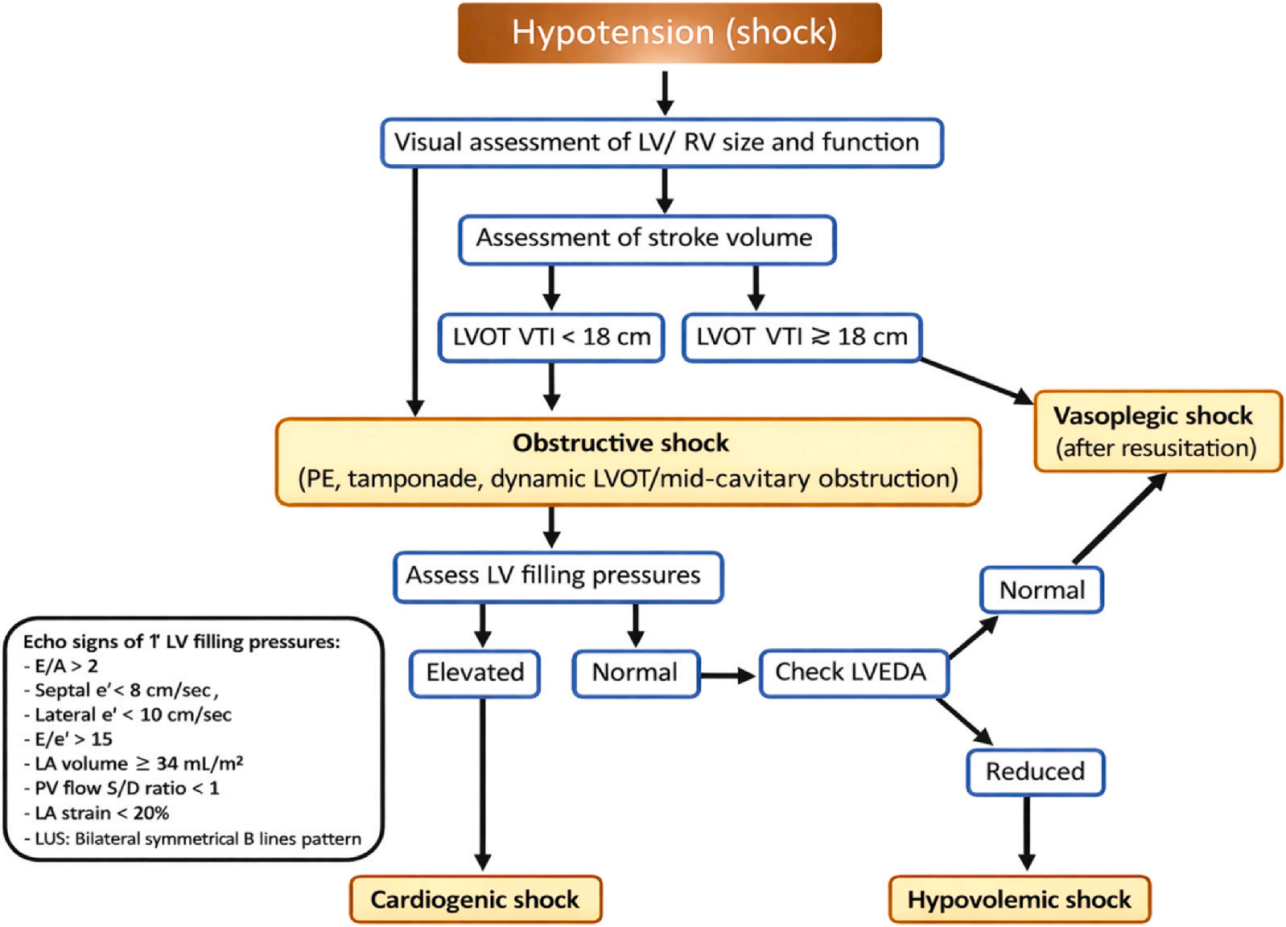
Proposed algorithm for the Echo-guided management of patients with hypotension. E/A, early-diastolic wave by transmitral pulsed-wave Doppler/late diastolic wave by transmitral pulsed-wave Doppler; LA, left atrial; LUS, lung ultrasound; LV, left ventricle; LVEDA, left ventricular end-diastolic area; LVOT, left ventricular outflow tract; PE, pulmonary embolism; PV, pulmonary veins; RV, right ventricle; TAPSE, tricuspid annular plane systolic excursion; tIVT, total isovolumic time; VTI, velocity time integral.

Adapted from Soliman-Aboumarie *et al*.^7^

Occurring in around 20% of Takotsubo patients or those with sepsis/hypovolaemia, this is characterised by a peak Doppler gradient greater than 30 mmHg. It is often precipitated by systolic anterior motion (SAM) of the mitral valve. Crucially, this condition can deteriorate with inotropic therapy, which accelerates flow and exacerbates the Venturi effect, drawing the mitral leaflet toward the septum. Recognition of dynamic LVOT obstruction is of critical importance in the acute and critical care setting, as it may present as an iatrogenic and potentially reversible cause of low cardiac output and haemodynamic instability. Notably, this condition may paradoxically deteriorate with escalation of inotropic therapy, which can exacerbate obstruction and further compromise forward flow (Fig. 2).

## POCUS in assessing hypoxaemia

POCUS provides a structured algorithm for hypoxaemia by integrating cardiac and pulmonary findings.

**Fig. 3:**
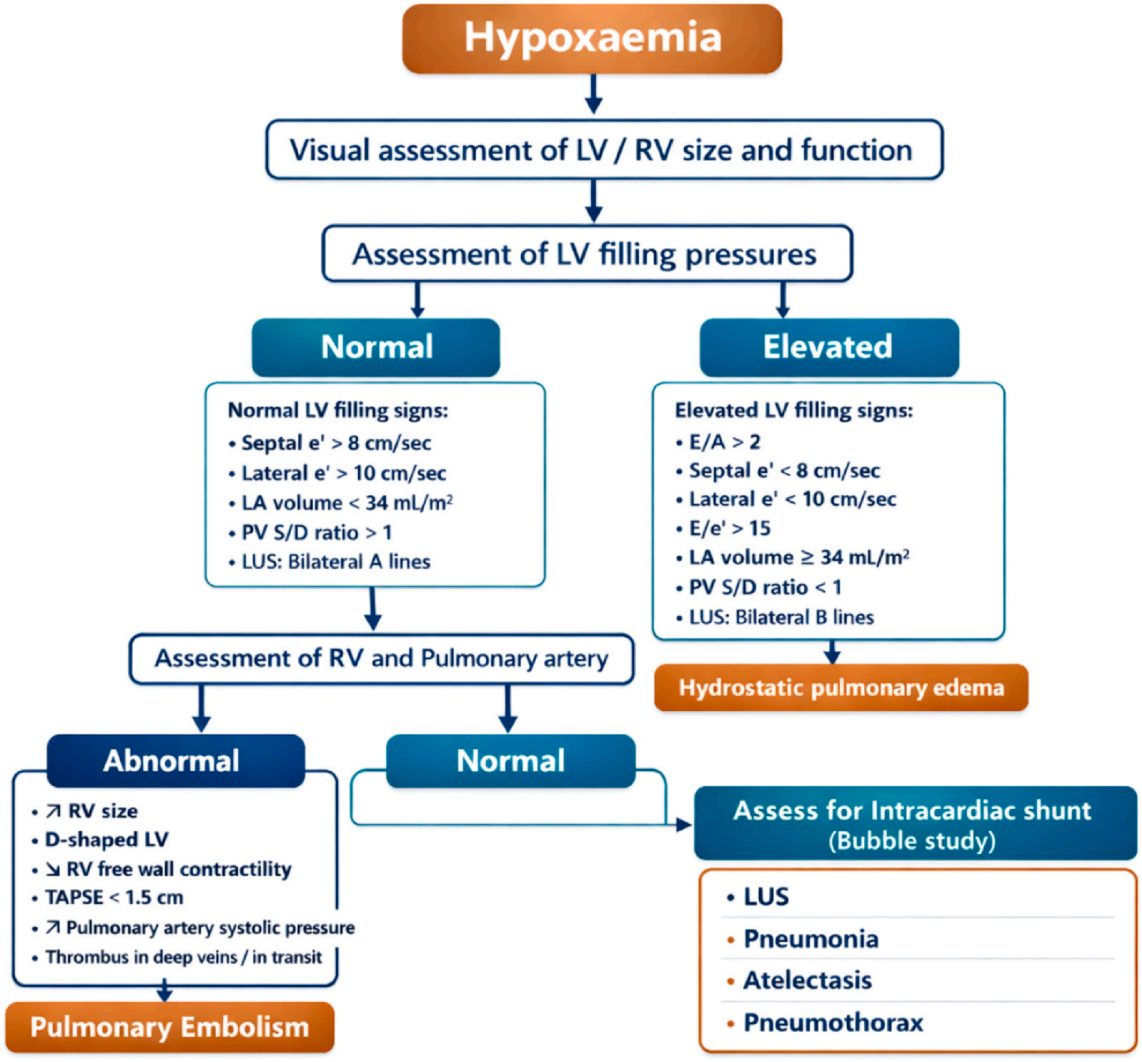
Proposed algorithm for the echo-guided assessment of patients with hypoxaemia. ASD, atrial septal defect; E/A, early-diastolic wave by transmitral pulsed-wave Doppler/late diastolic wave by transmitral pulsed-wave Doppler; E′, mitral annular velocity by tissue Doppler imaging; LA, left atrial; LUS, lung ultrasound; LV, left ventricle; PFO, patent foramen ovale; PV, pulmonary veins; RV, right ventricle; TAPSE, tricuspid annular plane systolic excursion.

Adapted from Soliman-Aboumarie *et al*.^7^

1. **Elevated filling pressures + bilateral B-lines:** suggests cardiogenic pulmonary oedema.
2. **Normal** f**illing pressures**+ bilateral**A-lines:** prompts evaluation for RV dilation or PE.
3. **Primary lung pathology:** identification of pneumonia, atelectasis, or pneumothorax.
4. **Intrapulmonary shunting:** Doppler interrogation can reveal deoxygenated blood bypassing ventilated alveoli, appearing as flow within pulmonary consolidations (Fig. 3).

## The POCUS pyramid: a proposed framework

The POCUS pyramid emphasises that haemodynamic assessment should not rely on a single organ. Instead, it integrates three pillars:

• **Echocardiogram:** assesses pump function and pressures.
• **Lung ultrasound:** validates filling pressures and identifies parenchymal disease.
• **Systemic venous assessment:** confirms the impact of right-sided loading via venous Doppler patterns.

**Fig. 4:**
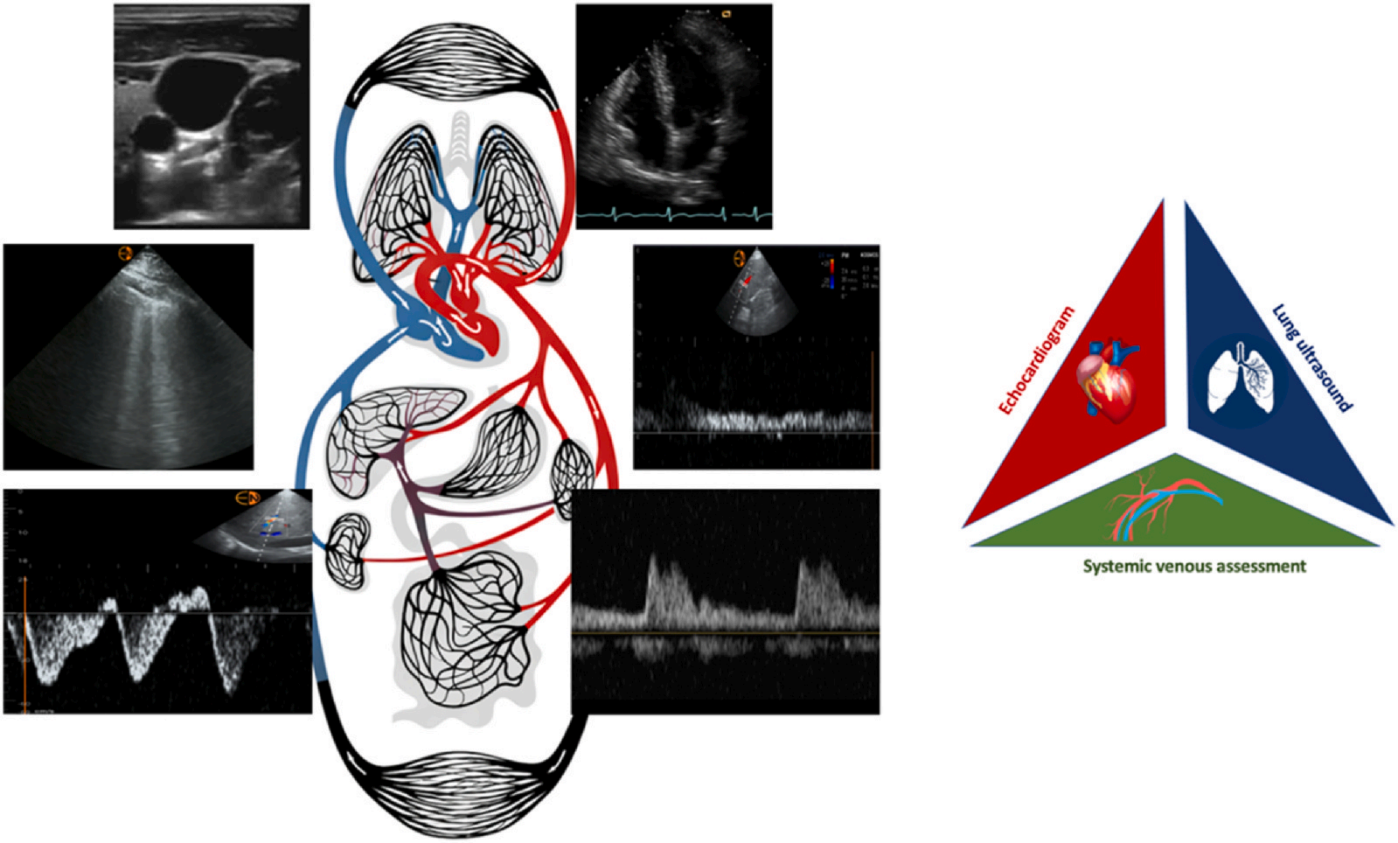
This central illustration summarises the POCUS pyramid which integrates the three pillars of haemodynamic POCUS (cardiac, lung and systemic venous congestion).

This integrative construct allows information to flow bidirectionally, transforming ultrasound into a dynamic bedside physiological model (Fig. 4).

### Summary

The evolution of POCUS over the last several decades has resulted in its essential and integral utility in the management of acute and critically ill patients. This CME review has summarised the role of POCUS and CCE when practiced by the appropriately trained treating physician, including indications, applications, implementation, and interpretation, while recognising potential pitfalls and challenges to ensure safe and effective bedside application.

## CRediT authorship contribution statement

**Hatem Soliman Aboumarie:** Writing – review & editing, Writing – original draft, Visualization, Resources, Project administration, Methodology, Formal analysis, Data curation, Conceptualization.

## Funding

This research did not receive any specific grant from funding agencies in the public, commercial, or not-for-profit sectors.

## Declaration of Competing Interest

The authors declare that they have no known competing financial interests or personal relationships that could have appeared to influence the work reported in this paper.
